# Effect of Co-Treatment of Olanzapine with SEP-363856 in Mice Models of Schizophrenia

**DOI:** 10.3390/molecules27082550

**Published:** 2022-04-14

**Authors:** Lingzhi Liang, Xia Ren, Junyi Xu, Yurong Ma, Yunlin Xue, Tao Zhuang, Guisen Zhang

**Affiliations:** 1 Department of Pharmacy, Jiangsu Ocean University, Lianyungang 222005, China; 2019220320@jou.edu.cn (L.L.); 2019220333@jou.edu.cn (X.R.); 2019220357@jou.edu.cn (J.X.); 2019220325@jou.edu.cn (Y.M.); 2021226083@jou.edu.cn (Y.X.); 2 Department of Biomedical Engineering, College of Life Science and Technology, Huazhong University of Science and Technology, Wuhan 430074, China

**Keywords:** olanzapine, SEP-363856, schizophrenia, synergistic, side effects

## Abstract

Olanzapine is a commonly used drug in the treatment of schizophrenia, but its clinical application has been restricted by metabolic-related side effects. In order to mitigate the weight gain side effects caused by olanzapine, other drugs with different targets were selected for combined use and evaluated in animal models of schizophrenia. SEP-363856 is a novel psychotropic agent which is under phase III clinical trials for schizophrenia treatment. The aim of the research was to evaluate whether co-administration of olanzapine and SEP-363856 exerts synergistic anti-schizophrenic effects in the apomorphine (APO)-induced climbing test, the MK-801-induced hyperactivity test, and the Morris water maze test, and therefore reduces the weight gain side effects induced by olanzapine. Through isobolographic analysis, the results showed a synergistic interaction in the climbing test; the experimental ED_30_ (3 mg/kg) was significantly smaller (*p* < 0.05) than the theoretical ED_30_ (5 mg/kg). Additionally, such potentiating effects appeared additive in the MK-801 challenge experiment. Co-treatment with an effective dose of olanzapine and a low dose of SEP-363856 reversed MK-801-induced cognitive impairment symptoms in mice. Moreover, combination treatment with olanzapine and SEP-363856 controls sustained weight gain in mice with chronic exposure to olanzapine. These results support further clinical trials to test the effectiveness of co-treatment of olanzapine and SEP-363856 for controlling symptoms and weight gain in patients with schizophrenia during antipsychotic treatments.

## 1. Introduction

Schizophrenia is a psychiatric disorder associated with the central nervous system, characterized by successive or repeated episodes of psychosis [1]. The main symptoms include positive symptoms (such as hallucinations, disordered thoughts), which are mainly caused by excessive dopamine (DA) in the limbic system of the midbrain, negative symptoms (psychomotor poverty), and cognitive deficits (such as memory deterioration and attention deficit disorder), which are caused by a lack of DA in the prefrontal cortex (PFC) [2,3]. Although a lot of research has been conducted on schizophrenia, its etiology and pathology have not been fully revealed [4]. This makes it extremely difficult to develop a therapy that can treat three symptoms of schizophrenia simultaneously. At present, the main treatment for schizophrenia is drug therapy. The first generation of drugs (typical antischizophrenic drugs) can calm agitated mental patients and produced sedation without impairing mental lucidity, but they can cause extrapyramidal motor disorders [5,6]. Second-generation (atypical antipsychotics) compared with typical antipsychotic drugs (haloperidol), they have a wide range of advantages, including partial relief of negative symptoms, a slight reduction in cognitive impairment, good efficacy, and high safety [7]. Some studies have shown that atypical antipsychotic drugs can relieve both positive and negative symptoms of schizophrenia [5,8,9]. However, metabolism-related side effects are generated after long-term usage of them, such as weight gain, diabetes, and heart metabolism disorders [10].

The atypical antipsychotic drug olanzapine, a thienobenzodiazepine derivative, is an antagonist of dopamine D_2_ receptors and is a commonly used drug for the treatment of schizophrenia [11]. Olanzapine is valid in the therapy of positive symptoms, also alleviates negative symptoms, and slightly reduces cognitive impairment, but it has the risk of weight gain, increased serum prolactin levels, and hyperglycemia [12]. In addition to its high affinity for dopamine D_2_, it also has high affinities for many neurotransmitter receptors such as serotonin 5-HT_2A_, 5-HT_2C_, histamine H_1_, and muscarinic M_1_ receptors [13]. Furthermore, evidence showed that histamine H_1_ receptors and 5-HT_2C_ receptors are involved in metabolic secondary actions of anti-psychotic drugs [14].

SEP-363856 was previously discovered in a medicinal chemistry effort utilizing a high-throughput mouse-behavior phenotyping platform, in combination with in vitro screening, aimed at developing a Non-D_2_ (anti-target) compound that was still effective in D_2_-receptor-based animal models [15]. SEP-363856 possesses a different mechanism of action from other drugs that simultaneously activate TAAR1 and 5-HT_1A_ receptors in vitro [15,16]. It has been found that SEP-363856 in the phencyclidine (PCP)-induced hyperactivity, prepulse inhibition (PPI), and PCP-induced deficits in social interaction demonstrated broad efficacy [15]. Currently, SEP-363856 is under in Phase III clinical trials [17], and shows great potential to treat both the positive and negative symptoms of schizophrenia, and there is no risk of weight gain and elevated serum prolactin levels [15]. Although the etiology and pathophysiology of schizophrenia remain unclear [18], substantial evidence links changes in neurotransmitters such as dopamine and serotonin to the onset of schizophrenia [19,20]. It has been found that TAAR1 is an important regulator of DA and the 5-HT system and is considered to be a significant target for the treatment of schizophrenia [19,21].

In clinical treatment, there are more and more cases where the best therapeutic effect has been achieved through the combination therapy of two or more anti-schizophrenia drugs. For example, olanzapine alone significantly alleviated only the positive symptoms of schizophrenia but not the negative symptoms, whereas the patient’s negative symptoms were notably improved after adding aripiprazole for combined treatment [22]. Another example is the combination of amisulpride and olanzapine in treatment-resistant schizophrenics [22]. In this study, side effects, including body weight gain and extrapyramidal motoric symptoms, were minimized with effective doses of amisulpride and olanzapine. Olanzapine has attracted much attention since it was put on the market, and it is also one of the most widely used and most effective drugs for the treatment of schizophrenia in the clinic. According to literature reports, the cost of olanzapine treatment for each patient in China is as high as USD 4233.31 through one year’s data statistics [23], which shows the market share of olanzapine is still relatively high. Although olanzapine is effective in clinical application, the overall clinical value of olanzapine is restricted. The aim of this research is to assess whether combined administration of olanzapine and SEP-363856 can enhance or achieve synergistic anti-schizophrenia effects against positive symptoms and cognitive disorder in schizophrenia, and hence reduce the weight gain side effects induced by olanzapine.

## 2. Results

### 2.1. Olanzapine and SEP-363586 Combinations Exhibited a Synergistic Effect in the Apomorphine-Induced Climbing Test

**Figure 1:**
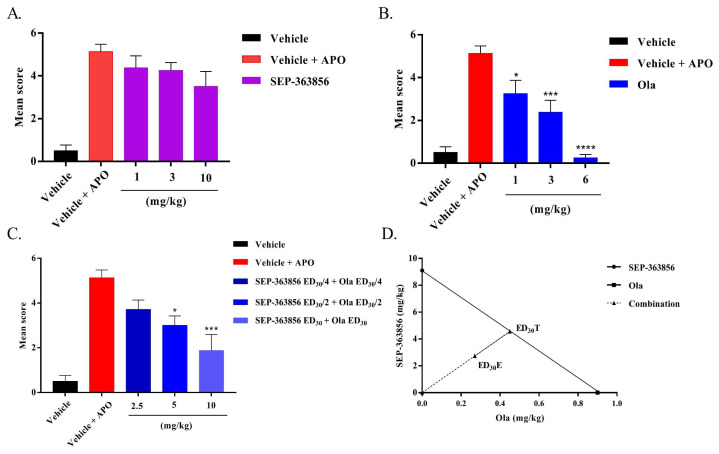
The effects of SEP-363856 and olanzapine on APO-induced climbing behavior in mice. Date expressed as mean ± S.E.M. (*n* = 8). (**A**) Effect of SEP-363856 (1, 3, 10 mg/kg, p.o.) and (**B**) olanzapine (Ola: 1, 3, 6 mg/kg, p.o.) on APO-induced climbing in mice. (**C**) Effect of Ola and SEP-363856 co-administration on APO-induced climbing in mice. (**D**) The equi-effective combination of SEP-363856 and Ola produces a synergistic effect, as it falls below the line of additivity. For this graph, the derived ED_30_ value of olanzapine was plotted on the abscissa and the ED_30_ value of SEP-363856 was plotted on the ordinate. (In alone administration compared with model group, * *p* < 0.05, *** *p* < 0.001, **** *p* < 0.0001).

Apomorphine (APO) is a dopamine receptor agonist [24]. Administration of apomorphine to mice produces a constant climbing behavior [25]. As shown in Figure 1A,B, SEP-363856 (1, 3, 10 mg/kg) did not significantly reverse APO-induced climbing behavior in mice, whereas a single oral administration of olanzapine (1, 3, 6 mg/kg) resulted in a dose-dependent inhibition of APO-induced climbing behavior (one-way ANOVA, *p* < 0.0001).

The ED_30_ values of SEP-363856 and olanzapine were calculated from their dose-response curves and were determined as 9.1 and 0.9 mg/kg, respectively. The combination of SEP-363856 and olanzapine exhibited a significant (*p* < 0.05) antipsychotic effect in the climbing test (Figure 1C). As shown in Figure 1C,D, the isobolographic analysis showed a synergistic interaction between these two drugs. The experimental ED_30_ of the combination was lower than the theoretical ED_30_ (3 mg/kg vs. 5 mg/kg, *p* < 0.05). In addition, the experimental ED_30_ is located below the isobologram that is the line corresponding to a purely additive interaction [26]. The interaction index (γ) for the SEP-363856–olanzapine combination was 0.6 (γ < 1). All these data suggest a synergistic interaction in mice following co-administration of SEP-363856 and olanzapine.

### 2.2. SEP-363856 Potentiates the Antipsychotic Properties of Olanzapine in MK-80-Induced Hyperactivity Test

MK-801, an NMDA receptor blocker, causes psychotic-like symptoms in normal humans that are similar to acute episodes of schizophrenia and exacerbate existing symptoms in schizophrenic patients [27]. Acute treatment with MK-801, which induces robust hyperactivity in rodents, is considered a valuable assay in preclinical research and is widely used to screen novel compounds for antipsychotic efficacy [15,28].

**Figure 2:**
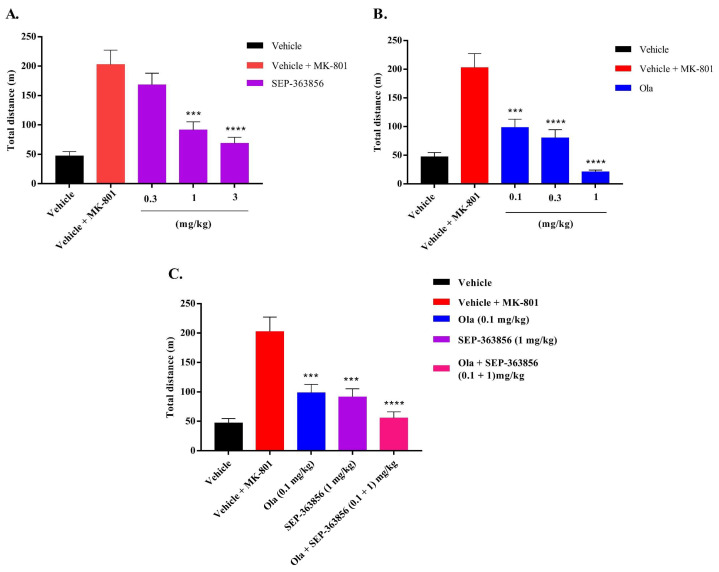
The effects of SEP-363856 and olanzapine on MK-801-induced hyperactive behavior in mice. Date expressed as mean ± S.E.M. (*n* = 8). (**A**) Effect of SEP-363856 (0.3, 1, 3 mg/kg, p.o.) on MK-801-induced hyperactive behavior in mice. (**B**) Effect of Ola (0.1, 0.3, 1 mg/kg, p.o.) on MK-801-induced hyperactive behavior in mice. (**C**) Effect of Ola (0.1 mg/kg) and SEP-363856 (1 mg/kg) co-administration on MK-801-induced hyperactive behavior in mice (compared with model group, *** *p* < 0.001, **** *p* < 0.0001).

A single oral administration of SEP-363856 (0.3, 1, and 3 mg/kg) and olanzapine (0.1, 0.3 and 1 mg/kg) dose-dependently inhibited hyperlocomotion induced by MK-801 (Figure 2A,B) [21,29,30], with ED_50_ values as 1.1 mg/kg and 0.1 mg/kg, respectively. SEP-363856 slightly reversed MK-801-induced locomotor activity at a lower dose (0.3 mg/kg) by ca. 17%, but reduced it by ca. 55% and 66% in medium and high doses (1 and 3 mg/kg), respectively (*p* < 0.001) (Figure 2A). Olanzapine at different doses (0.1, 0.3, and 1 mg/kg) all inhibited MK-801-induced locomotor activity by ca. 51%, 60%, and 90%, respectively (*p* < 0.001) (Figure 2B).

Co-treatment with the ED_50_ of SEP-363856 (1.1 mg/kg) and olanzapine (0.1 mg/kg) decreased the MK-801-evoked locomotor hyperactivity by ca. 73% (*p* < 0.0001) (Figure 2C). This enhancement effect appears to be additive in the MK-801 experiment. These observations suggest that SEP-363856 as an add-on treatment to current atypical antipsychotics can optimize therapeutic efficacy [21].

### 2.3. Combined Treatment of SEP-363856 and Olanzapine Reversed MK-801-Induced Cognitive Impairment Symptoms in Mice

The Morris water maze (MWM) test is used to test animals for memory performance [31]. This approach has become one of the “gold standards” for testing memory disorders in behavioral neuroscience [32].

**Figure 3:**
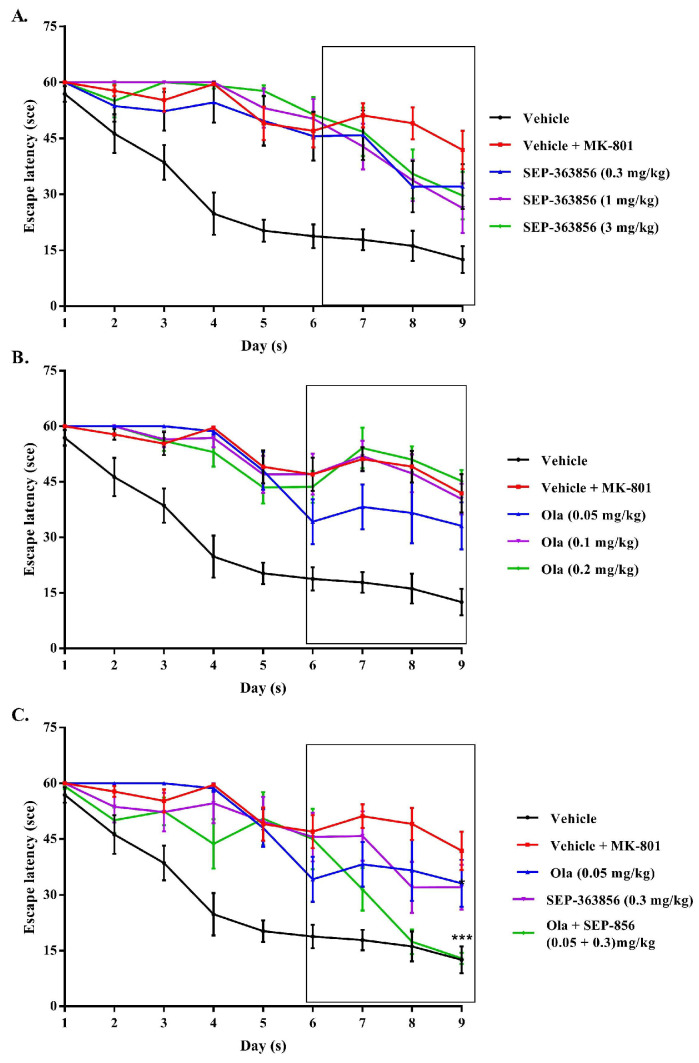
The effects of SEP-363856 and olanzapine on MK-801-induced memory impairment in Morris water maze behavior in mice. Date expressed as mean ± S.E.M. (*n* = 8). (**A**) Effect of SEP-363856 (0.3, 1, 3 mg/kg, p.o.). (**B**) Ola (0.05, 0.1, 0.2 mg/kg, i.p.), and (**C**) co-administration of Ola (0.05 mg/kg) and SEP-363856 (0.3 mg/kg) on MK-801-induced memory impairment in Morris water maze behavior in mice. The black box represents the period of treatment with olanzapine, SEP-363856, or their combination. (In the co-administration group, the ninth day, *** *p* < 0.001, model group vs. Ola + SEP-363856).

In the MWM experiments, compared with the vehicle group, there was an increased escape latency observed in MK-801-treated mice beginning from day 4 to day 9. Mice treated with saline or purified water, MK-801 (0.1 mg/kg), MK-801 (0.1 mg/kg) with SEP-363856 (0.3, 1, 3 mg/kg) (Figure 3A), or MK-801 (0.1 mg/kg) with olanzapine (0.05, 0.1, 0.2 mg/kg) (Figure 3B) took different amounts of time to find a platform in a water maze experiment. In the search session, the model mice took significantly more time to find the platform. The escape latency on days 6 to 8 was shortened in olanzapine-treated mice (0.05 mg/kg), but olanzapine (0.1 and 0.2 mg/kg) treatment did not affect the MK-801-treated mice. However, SEP-363856 (0.3, 1, 3 mg/kg) can reduce the escape latency compared with the model group. Co-treatment with an effective dose of olanzapine (0.05 mg/kg) and a low dose of SEP-363856 (0.3 mg/kg) reversed the cognitive impairment evoked by MK-801 (0.1 mg/kg) in this test. Two-way ANOVA indicated that the potentiating effect was significant when compared with the model group (*p* < 0.001). The escape latency on days 7 and 9 was decreased in olanzapine (0.05 mg/kg) and SEP-363856 (0.3 mg/kg)-treated mice and this difference was significant on day 9 (*p* < 0.05). (Figure 3C).

### 2.4. Co-Administration of Olanzapine and SEP-363856 Protects against Olanzapine-Induced Weight Gain

**Figure 4:**
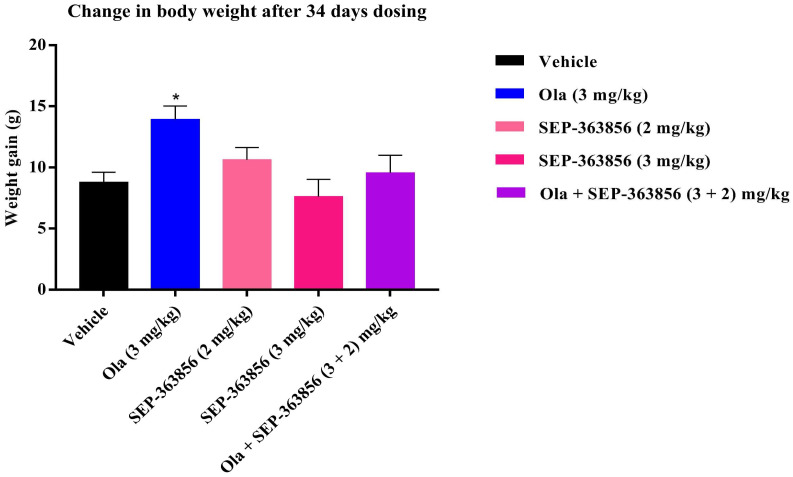
The effects of olanzapine (3 mg/kg, p.o.) and SEP-363856 (2, 3 mg/kg, p.o.) given alone or in combination with olanzapine (3 mg/kg) and SEP-363856 (2 mg/kg) on body weight gain over 34 days in female mice. Date expressed as mean ± S.E.M. (*n* = 8). (* *p* < 0.05 vs. vehicle group).

One major side effect of olanzapine is weight gain, which increases the risk of cardiovascular adverse events and diabetes, reduces the quality of life, and may interrupt treatment [28]. To assess the effect of the combination on weight gain side effects, we investigated the effect of combination treatment with olanzapine and SEP-363856 on body weight in long-term olanzapine-treated female mice. In mice which received daily treatment with SEP-363856 (2 and 3 mg/kg) for 34 days, there were no significant differences in body weight gain as compared with the vehicle. Conversely, mice treated daily with olanzapine (3 mg/kg, p.o.) experienced significant increases in weight compared with vehicle-treated animals (*p* < 0.05). However, co-treatment with a low dose of SEP-363856 (2 mg/kg, p.o.) prevented olanzapine (3 mg/kg, p.o) from causing weight gain (Figure 4). Overall, the SEP-363856 has a remarkable profile in concomitantly potentiating the antipsychotic effects of olanzapine while minimizing its metabolic-related adverse effects.

## 3. Discussion

Based on the present results, combined administration of olanzapine and SEP-363856 produced synergistic anti-schizophrenia effects in an APO-induced climbing experiment in mice, with a better result compared with single administration of these drugs. In the climbing model, APO is used to screen both classical and atypical agents as a tool drug [24]. At higher doses, APO stimulates motor activity and induces climbing behavior, mainly due to activation of postsynaptic D_1_ and D_2_ receptors [33]. TAAR1 is a G-protein-coupled receptor and is mainly expressed in the ventral tegmental area, the limbic system, the dorsal raphe, and the basal ganglia [34,35,36]. The activation of the TAAR1 receptor can increase intracellular cyclic adenosine monophosphate (cAMP) levels and enhance the effect of dopamine receptors in the mouse brain after administration of APO, which may lead to coactivation of different receptors and cause mice to keep climbing. Previous reports found that APO showed binding activity with TAAR1 in animals (mice, rats, monkeys, etc.), with the highest binding affinities observed in the mice and rats [33], which may be one reason for the ineffectiveness of SEP-363856 in the climbing test. In addition to its favorable pharmacokinetic profile, SEP-363856 also lacks D_2_ receptor occupancy, so this may also be a reason for its poor efficacy in climbing models [15]. However, it has a synergistic effect when used in combination with olanzapine, which may be due to the interaction between TAAR1 receptors, D_2_ receptors, and 5-HT_1A_ receptors in DA neurons in the ventral tegmental area (VTA) and 5-HT neurons in the dorsal raphe nucleus (DRN), respectively, thus changing their pharmacological properties [37,38]. In the MK-801-induced hyperactivity model, SEP-363856 and olanzapine can attenuate the hyperactivity induced by MK-801 in a dose-dependent manner [21]. In addition, we also found that the combined administration of olanzapine (0.1 mg/kg) and SEP-363856 (1.1 mg/kg) attenuated the hyperactive behavior of mice induced by MK-801, and was more effective than the single administration. This may be because atypical antipsychotics can enhance the anti-schizophrenic effect by indirectly acting on TAAR1 [21]. Perhaps in some areas of the brain, SEP-363856 and the olanzapine change brain activity in different ways, and the exact mechanism needs to be further explored. According to these two models of positive symptoms of schizophrenia, it showed that SEP-363856 may enhance the effects of olanzapine during combined administration. TAAR1 agonists are effective in models based on increased DA activity or reduced NMDA activity [21].

Cognitive impairment is believed to result from hyperfunctioning of the prefrontal cortex (PFC) which is a brain structure that plays an important role in working memory and actionability [39,40,41,42]. In the water maze test induced by MK-801, repeated injection of MK-801 also impairs learning and memory functions in the rat hippocampus [43,44]. However, in our experiment, only the lowest dose of olanzapine (i.p.) had the effect of reversing cognitive impairment. According to previous reports, olanzapine may have neuroprotective effects and these effects might be related to cognitive functions [43]. However, when the SEP-363856 was administered orally, three doses (0.3, 1 mg/kg and 3 mg/kg) all had a slight effect on relieving cognitive impairment. Although the mechanism of SEP-363856 action has not been fully elucidated, in vitro and in vivo pharmacology data suggest that agonism at both TAAR1 and 5-HT_1A_ receptors contributes to its efficacy [15]. Co-treatment with an effective dose of olanzapine (0.05 mg/kg) with a low dose of SEP-363856 (0.3 mg/kg) change the cognitive impairment evoked by MK-801 (0.1 mg/kg), showing the effect of improving cognitive impairment. These results show that the combined use of TAAR1 agonists and atypical antipsychotic drugs not only promotes the executive ability of rodents but also implies the potential benefits of reducing cognitive impairment in schizophrenia, which may provide a new idea for the treatment of cognitive impairments in schizophrenia in the future.

Antipsychotics usually bind to various neurotransmitters in the brain. Some of them, such as M_C_4r, 5-HT_2C_, and H_1_ receptors, are related to metabolism [14,45]. Therefore, although achieving therapeutic effects, there will also be side effects of weight gain when achieving their therapeutic effects. In rodent brains, the hypothalamic nucleus, dorsal raphe nucleus (DRN), and nucleus tractus solitarius control food intake and energy balance [21,43]. In addition to being expressed in the nervous system, TAAR1 is also expressed in peripheral organs that control energy balance and food intakes, such as the pancreas, stomach, and intestine [24]. When TAAR1 is activated in these organs, it may regulate digestion and food absorption, and insulin is secreted to counteract the metabolic disorders caused by olanzapine [46]. In the side-effects experiment on weight gain, olanzapine (3 mg/kg) caused significant weight gain in mice after long-term administration, whereas the same dose of SEP-363856 did not cause weight gain after long-term administration. On the contrary, combined administration prevented excessive eating caused by olanzapine to some extent, which may be due to the coactivation of TAAR1 in the central nervous system and peripheral organs. Therefore, there was no significant increase in body weight after long-term administration.

## 4. Materials and Methods

### 4.1. Animals

Adult male and female ICR mice (22 ± 4 g) were obtained from Pizhou Oriental Breeding Co., Ltd. (Xuzhou, China). The animals were housed under standardized conditions for temperature (21 ± 3 °C), humidity (50 ± 20%), and a 12/12 light/dark (lights on at 8 a.m., lights off at 8 p.m.) cycle. All mice had ad libitum access to food and tap water. Mice were assigned to different experimental groups randomly (*n* = 8), each kept in a separate cage. All efforts were made to minimize animal suffering and to reduce the number of animals used. All the experimental protocols were approved by the Ethics and Experimental Animal Committee of the Jiangsu Ocean University (Project identification code: 202100014).

### 4.2. Drugs and Treatments

Olanzapine API (purity > 99%) was obtained from Shanghai Aladdin Bio-Chem Technology Co., Ltd. (Shanghai, China) and dissolved in a minimum volume of glacial acetic acid before adding purified water, and pH was adjusted to 6.0 with 0.1 M NaOH. SEP-363856 API (purity > 95%) was synthesized in our laboratory, and dissolved in purified water. Apomorphine (APO) was obtained from Sigma-Aldrich (Shanghai, China) Trading Co., Ltd., and dissolved in saline (with 0.1% ascorbic acid). Dizocilpine maleate (MK-801) was obtained from Shanghai Macklin Biochemical Co., Ltd. (Shanghai, China) and dissolved in saline before use.

### 4.3. Animal Studies

#### 4.3.1. Apomorphine-Induced Climbing Behavior

Before the beginning of the experiment, the mice were put into cages (12 cm in diameter, 14 cm in height) for an hour, and then were orally dosed with vehicle or doses of the SEP-363856 (1, 3, 10 mg/kg) and olanzapine (1, 3, 6 mg/kg). Immediately after that, each mouse was put into a small climbing cage. According to the pharmacokinetic properties of SEP-363856 and olanzapine, APO (1 mg/kg, s.c.) was given in SEP-363856 group mice 5 min after administration, and APO (1 mg/kg, s.c.) was given in the olanzapine group after 60 min. When used in combination, SEP-363856 was given 55 min after olanzapine, APO (1 mg/kg) was given 5 min later, and mice were observed for climbing behavior at 10, 20, and 30 min post-dose [3]. The climbing behavior was scored as follows: 3,4 paws on the cage floor = 0 score; 2 and 3 paws on the cage floor = 1 score; 4 paws on the cage floor = 2 score [3].

Isobolographic analysis was used to assess drug–drug interactions [47,48]. The theoretical effective dose (ED_30_T) was calculated based on the dose-response curves of olanzapine and SEP-363856 [45]. To obtain the experimental ED_30_ (ED_30_E) for both drugs, each group was treated with the following dose: (1) SEP-363856 ED_30_ (9.1 mg/kg) + olanzapine ED_30_ (0.9 mg/kg); (2) SEP-363856ED_30_/2 (4.55 mg/kg) + olanzapine ED_30_/2 (0.45 mg/kg); (3) SEP-363856ED_30_/4 (2.275 mg/kg) + olanzapine ED_30_/4 (0.225 mg/kg), as shown in Table 1. The value of the theoretical ED_30_ (ED_30_T) was compared with the experimental ED_30_ (ED_30_E) to determine the interaction type (synergistic, additive, or antagonistic) and differences were considered (*p* < 0.05). Additionally, the (γ) represents the interaction index with synergistic interaction: γ < 1; antagonistic interaction: γ > 1; additive interaction: γ = 1 [49].

#### 4.3.2. MK-801-Induced Hyperactivity

Before the experiment, the mice were habituated in the open field test box (33 cm × 33 cm × 33 cm) for 30 min and orally given vehicle or increasing doses of olanzapine (0.1, 0.3, 1 mg/kg) and SEP-363856 (0.3, 1, 3 mg/kg). After 30 min, the animals were challenged with MK-801 (0.3 mg/kg, i.p.), and the locomotor activity of each animal was recorded for 60 min. When used in combination, a dose less than the ED_50_ of olanzapine (0.1 mg/kg) and SEP-363856 (1 mg/kg) was used, and both drugs were given at the same time. MK-801 (0.3 mg/kg, i.p.) was given 30 min later, and mice were placed in the activity boxes and the total distance of their activities was recorded for 60 min [3].

#### 4.3.3. Morris Water Maze Test

**Figure 5:**
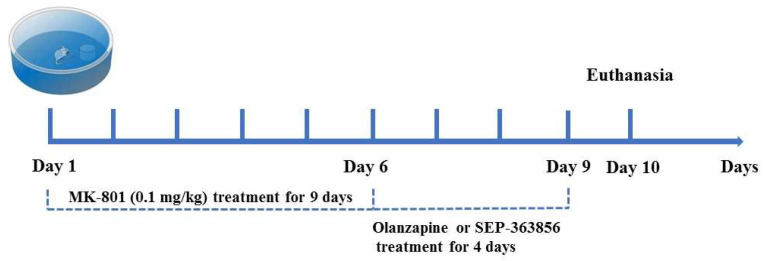
Schematic diagram of experiment schedule. MK-801 (0.1 mg/kg) was administered 20 min before behavioral testing for a total of 9 days. Following 6 days of treatment with MK-801, olanzapine and SEP-363856 were administered 40 min and 10 min before MK-801 administration, respectively, for a total of 4 days. Spatial memory performance was measured in a Morris water maze test over 9 days (4 trials/day).

To determine the learning and memory of compounds in vivo, the effects of different doses of compounds in the Morris water maze (MWM) model were assessed. A circular swim pool measuring 1200 cm in diameter and 55 cm in height was filled to a depth of 40 cm with water (maintained at 25 ± 2 °C). The maze was divided into four quadrants, and three equally spaced points served as starting positions around the edge of the pool. A circular escape platform (10 cm diameter and 12 cm high) was located 1 cm below the water surface. ANY-maze software was used to record the escape latency of the mouse automatically. The mouse was put into the water from a fixed quadrant of the circular pool, four times a day, and for 60 s each time. After reaching the platform, the mouse was allowed to stay on the platform for 5 s and then moved to a drying cage where it stayed for 15 min before being returned to the original cage. If the mouse did not find the platform within 1 min, it was allowed to remain on the platform for 30 s. MK-801 (0.1 mg/kg) was administered 20 min before finishing the behavioral testing for a total of 9 days [41]. Following 6 days of treatment with MK-801, either olanzapine (0.05, 0.1, 0.2 mg/kg, i.p.) or SEP-363856 (0.3, 1, 3 mg/kg, p.o.) was administered 40 min before or 10 min before the administration of MK-801, respectively, for a total of 4 days (Figure 5). The effective dose of olanzapine and the minimum dose of SEP-363856 were used in combination, and the average escape latency of mice within 60 s was recorded.

#### 4.3.4. Weight Gain Test

In the previous preliminary experiment, it was shown that olanzapine (3 mg/kg) could increase the weight of mice, so olanzapine (3 mg/kg) was used for the experiment. Mice were orally dosed with vehicle or doses as follows: SEP-363856 (2, 3 mg/kg, p.o.) and olanzapine (3 mg/kg, p.o.), administered once a day for 34 consecutive days. It was ensured that mice drank and ate normally every day and the weight of mice was tested every day [3].

### 4.4. Statistical Analysis

Statistical analyses were performed using the commercially available software GraphPad Prism v7.0. All data obtained are expressed as the mean ± standard error of the mean (SEM). Multiple group comparisons were assessed with one- and two-way analysis of variance (ANOVA). The differences between groups were significant if the *p*-value was below 0.05.

## 5. Conclusions

In summary, our results showed that olanzapine combined in treatments with SEP-363856 could ameliorate the memory deficits in MK-801-induced schizophrenia in mice and enhance the anti-schizophrenia effect of positive symptoms in a synergistic manner. We found that co-treatment using olanzapine with SEP-363856 significantly reduced weight gain side effects induced by olanzapine through the long-term treatment course. This study provides further evidence to support clinical research to test the effectiveness of co-treatment with olanzapine and SEP-363856 for controlling the positive symptoms, cognitive impairment, and the weight gain side effect in schizophrenia with long term antipsychotic treatment.

## Figures and Tables

**Table 1:** ED_30_ used in the interaction between SEP-363856 and olanzapine before administration of APO.

Dose (mg/kg Body Weight)				Inhibition Ratio (%)
Group (*n* = 8)	SEP-363856	Olanzapine	Total	
(1)	9.1	0.9	10	63%
(2)	4.55	0.45	5	41%
(3)	2.275	0.225	2.5	27%

## Data Availability

The raw data supporting the conclusions of this article will be made available by the authors, without undue reservation.

## Sample Availability

Samples are not available.
